# Preceptorship Barriers From the Preceptors’ and Preceptees’ Lens: A Qualitative Study

**DOI:** 10.1155/nrp/4391429

**Published:** 2026-07-30

**Authors:** Adnan Innab, Juliana Linnette D′Sa, Sahar Alshamrani, Salha Alshamari, Amani Awaji, Nawdhaa Alotaibi, Wejdan Shaqiqi

**Affiliations:** ^1^ Nursing Administration and Education Department, College of Nursing, Riyadh, Saudi Arabia; ^2^ Maternal and Child Health Nursing Department, College of Nursing, Riyadh, Saudi Arabia; ^3^ King Saud Medical City, Riyadh, Saudi Arabia, ksmc.med.sa; ^4^ College of Nursing, King Saud Bin Abdulaziz University for Health Sciences, Riyadh, Saudi Arabia, ksau-hs.edu.sa; ^5^ King Abdullah International Medical Research Center, Riyadh, Saudi Arabia, kaimrc.med.sa; ^6^ Ministry of the National Guard-Health Affairs, Riyadh, Saudi Arabia

**Keywords:** clinical practice, nursing, preceptee, preceptor, preceptorship

## Abstract

**Background:**

Preceptorship is a cornerstone of clinical teaching in undergraduate nursing programs in several countries, including Saudi Arabia. However, the common challenges faced by both preceptors and preceptees have not been explored.

**Aim:**

To explore the preceptors’ and preceptees’ perceptions toward barriers faced during preceptorship program.

**Methods:**

A qualitative descriptive design using Braun and Clarke’s six‐step thematic analysis was adopted, and online semistructured interviews were conducted for each participant from March to June 2022. A purposive sample of 13 participants (eight preceptors and five preceptees) was recruited from hospitals in different regions of Saudi Arabia. To ensure scientific rigor, the study’s credibility, confirmability, and transferability were determined.

**Results:**

Two major themes emerged: (1) challenges of the preceptorship program (i.e., language barrier, lack of readiness to practice, lack of time, and lack of role preparation and direction and (2) strategies for improving preceptorship programs (i.e., having an organized preceptorship program and having organizational support).

**Conclusion:**

Preceptorship plays a vital role in helping nursing students develop clinical knowledge, confidence, and practical skills during their transition into professional practice. This study identified key barriers affecting effective preceptorship, including language challenges, limited readiness for practice, insufficient time, and unclear role preparation for both preceptors and preceptees. Strengthening preceptorship programs through structured organization, adequate staffing, proper preceptor support, and standardized training strategies can enhance clinical learning experiences and improve educational outcomes.

**Reporting Method:**

The Consolidated Criteria for Reporting Qualitative Research checklist was followed.

## 1. Background

Preceptorship has become the leading approach to clinical teaching in undergraduate nursing programs in several nations, including Saudi Arabia. It is effective in developing competent and positively influences quality of care, which is imperative in enhancing healthcare quality [1]. Preceptorship is a structured period of clinical training during which experienced nurses provide support and guidance to students, interns, or novice nurses to enhance their clinical competence and confidence [2].

Nurse preceptors play a crucial role in sharing their knowledge and skills with preceptees to integrating theory with practice and help them achieve their learning outcomes [3]. They facilitate the educational activities of their preceptees and enhance their active participation as members of the health team. Preceptors are often licensed nurses who undergo orientation programs and training as provided by their organizations [4]. They are clinically trained nurses working in healthcare facilities where nursing students and interns are assigned for their clinical placement/internship [4].

In Saudi Arabia, preceptorship is a critical teaching component of the nursing curriculum. The undergraduate nursing program consists of eight semesters, which include clinical placements in hospital settings for various subjects that require students to perform certain tasks under supervision. After completion, a 12‐month internship program is mandatory to obtain the nursing license. It can assist interns in developing the required competencies and career exploration, as they transition into their roles as professional nurses [5]. Preceptorship teaching is of paramount importance in an internship program, for interns to develop their knowledge, competencies, and proficiency under the supervision of competent nurses. A previous study on undergraduate nursing interns in Saudi Arabia found that most nursing interns in a Saudi study perceived preceptorship as a constructive experience that enhanced their competencies in clinical settings [4]. A similar study among newly hired nurses in Saudi Arabia indicated that participation in a 90‐day preceptorship program enhanced the novice nurses’ self‐efficacy [6].

Despite preceptorship’s benefits, previous research has shown that preceptors and preceptees may not be ready for their respective roles [7, 8], leading to several challenges, which ultimately hinder the achievement of its intended objective. Nurses may not be adequately trained for their roles as preceptors [9] and may experience conflict between their role as nurses and preceptors [10]. In addition, factors such as language barriers [11] and lack of preceptors’ availability for one‐on‐one interactions can negatively influence the experiences of both preceptors and preceptees [12]. A study exploring expatriate professional nurses’ experiences of preceptorship in Saudi Arabia highlighted that some preceptors were not welcoming, whereas others lacked sufficient time to teach or viewed preceptees as an additional burden alongside their workload [13].

Identifying the challenges of preceptorship from the perspectives of both nurses and students is essential to address the widening gap between clinical education and real‐world practice, directly impacting staff retention and patient care. Despite its significance, a few studies have explored the experiences of preceptorship programs in Saudi Arabia [4, 14]. Additionally, there is a dearth of research on preceptorship from the perspectives of both preceptors and preceptees. Most of the published studies on preceptorship examined challenges either from the perspective of students or interns [15] or interns and faculty [16]. Identifying common obstacles from both preceptors’ and preceptees’ perspectives bridges differing viewpoints and facilitates addressing them most effectively and efficiently. This can ultimately enhance the overall quality of the learning and mentoring experience, reduce burnout for preceptors, and alleviate anxiety for new hires, fostering a safer, more effective learning environment.

Given the significance of preceptorship in preparing interns for their professional careers, our aim in this study was to explore preceptorship program barriers from both preceptors’ and preceptees’ perceptions. This study’s data will help educators understand the challenges faced by nursing interns and preceptors in clinical settings and identify strategies to improve preceptorship programs.

## 2. Material and Methods

### 2.1. Study Design

A qualitative descriptive design using Braun and Clarke’s six‐step thematic analysis [17] was adopted to explore preceptorship programs barriers from preceptors’ and preceptees’ perceptions. This design was used to gain in‐depth knowledge about participants’ experience and recognize the similarities and differences among participants involved in preceptorship programs. Such a design ensures that the data analysis remains authentic to participants’ narratives and that the researchers’ views are transparent. As this study involved participants from different hospitals, the interviews were conducted virtually. The semistructured interview questions are presented in Table 1. We adhered to the Consolidated Criteria for Reporting Qualitative Research checklist to ensure rigor.

**TABLE 1:** Interview guide.

Target	Questions
Introductory questions	1. What do you think of preceptorship programs?

Key questions for preceptors	2. Tell me about your experience of being a preceptor. 3. What challenges have you faced in your role as a preceptor? 4. How did you overcome these challenges? 5. From your point of view, what do you think should be done to improve the current state of preceptorship programs?

Key questions for nursing preceptees	2. Tell me about your experience of being a preceptee. 3. What challenges have you faced in your role as a preceptee? 4. How did you overcome these challenges? 5. As a nursing intern, what do you think should be done to improve the current state of preceptorship programs?

Closing questions	6. Are there any additional points that you would like to add?

Supplementary questions	1. Tell me more about it. 2. Can you give me some examples? 3. Can you explain a little more?

### 2.2. Participants and Setting

Purposive sampling was used for recruitment. The inclusion criteria for preceptors were (1) registered nurses with at least 2 years of experience in a clinical setting, working as preceptors for interns in Saudi Arabia at the time of the study, and (2) provision of informed consent. The inclusion criteria for preceptees were (1) interns completed more than 3 months of nursing internship, (2) experience training in clinical settings in Saudi Arabia, and (3) provision of informed consent. The exclusion criteria for preceptors and preceptees were nurse educators working in academic settings/outpatient clinics and interns who were no longer practicing nursing skills in inpatient departments/had completed their internship year, respectively. As the interview questions were aimed to be delivered in English only, both preceptors and preceptees who did not have adequate English proficiency were excluded.

Sixteen participants were initially recruited. However, three participants (one preceptor and two interns) opted out because the interviews were audio‐recorded and they were concerned about the repercussions for their jobs or studies. The final sample included 13 participants (eight preceptors who are registered nurses and five preceptees who are nursing interns). All participants were recruited from public hospitals in different regions of Saudi Arabia (4 Western Region, 4 Northern Region, 3 Central Region, and 2 Eastern Region). Most participants were female (*n* = 12).

### 2.3. Data Collection Procedure

Data were collected between March and June 2022. An announcement about the study was sent directly to head nurses in different departments after obtaining permission from the nursing director of each hospital. Individuals who agreed to participate in this study were invited to virtual meetings. Semistructured interviews (comprising open‐ended questions) were conducted separately with each participant by three trained members of the research team (Sahar Alshamrani, BA, and Salha Alshamari). To ensure consistency, the interviewers were requested to follow the interview guide (Table 1), created following a thorough examination of pertinent literature and collaborative discussions among the research team members. In addition, one researcher interviewed interns, and two interviewed nurses.

**FIGURE 1:**
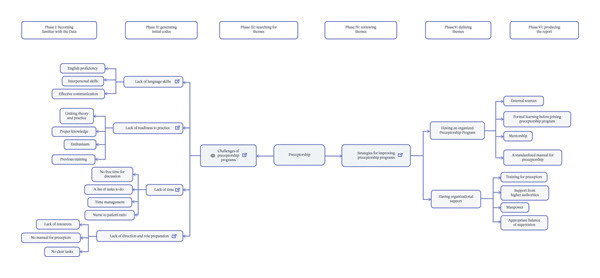
The process of categorizing the raw interview data to generate the themes using NVivo 10.1 software.

Interviews were conducted virtually using Zoom. To support a clear audit trail and protect participants’ confidentiality, several procedures were followed. Each participant received a secure meeting link to join the interview. At the beginning of the session, the researcher verbally reconfirmed informed consent, and this confirmation was recorded. Participants were also informed that their participation is voluntary and their information would remain confidential. Participants could withdraw from the study at any time during the interview without any consequences. All recordings and transcripts were coded using a systematic coding process (Figure 1).

The interviewers were requested to adhere to the time limit of the interview. Each interview lasted 30–45 min, with an average of 32 min. Interviews were audiotaped and transcribed verbatim after the interview. The research team stopped collecting data after reaching data saturation, when new information no longer offered fresh insights related to the research questions. Data saturation was reached after interviewing 5 interns and 8 nurses (13 in total). Interviews with preceptors were coded as “PI,” while interviews with intern nurses were coded as “NPI.” All digital files were then transferred to a password‐protected and encrypted drive, and any cloud‐based files were deleted. Access to the data was limited to the research team only, ensuring confidentiality, transparency, and data integrity.

### 2.4. Data Analysis

NVivo 10.1 software was used to code and analyze the data and document the data analysis. The researchers followed Braun and Clarke’s six‐step thematic analysis: becoming familiar with the data by reading and rereading the dataset, creating initial codes, searching for themes, reviewing themes, defining themes, and writing reports [17, 18]. During the data analysis, some codes were modified, and new codes were added by two researchers (Adnan Innab and Juliana Dsa). The research team took part in several discussions regarding the assigned codes to verify the accuracy of the coding. Figure 1 describes the process of categorizing the raw interview data to generate the themes.

The subsequent step involved identifying themes by clustering similar or frequently occurring codes and further examining them to determine whether the codes fit together into a theme. In this manner, several themes that described patterns significant to the data were developed, and subthemes were identified. The themes and subthemes were then reviewed against the aim and dataset, and the report was written. Two researchers (Adnan Innab and Juliana Dsa) reviewed the themes and registered their findings. The report contained excerpts from the transcripts.

### 2.5. Rigor

To ensure scientific rigor, the study’s credibility, confirmability, and transferability were determined in line with prior research [19]. Participants were informed that they could ask for clarifications if they did not understand the questions. Credibility was verified by audio recording the semistructured interviews and discussions, engaging with the data and brainstorming, and feedback within the research team. We used the same interview guide and documentation process to confirm the qualitative data. Confirmability was achieved by sending the transcripts to another member of the research team who independently coded the data and compared their coding with that of the first coder. Moreover, the themes report was sent to participants for their feedback. To achieve transferability, a rich description of research methods and demographic characteristics was provided, along with transcriptions of participants’ responses; this ensured that the results reflected participants’ experiences.

### 2.6. Ethical Statement

Institutional Review Board approval was obtained from the Ministry of Health, Saudi Arabia [H‐01‐R‐012], before data collection. This study was conducted according to the principles of the Declaration of Helsinki. Informed consent was obtained from the participants, who also indicated their agreement to audiotape the interviews. Participants were informed that the interviews would be conducted in English and they had the right to withdraw at any time without consequences. Confidentiality was maintained throughout the interviews. Participants were asked only to clarify the type of hospital (public or private) without providing the name and to use pseudonyms for themselves. Audio recordings were securely handled by the research team and destroyed after the publication of the research findings.

## 3. Results

### 3.1. Theme 1: Challenges of Preceptorship Programs

Both preceptors (nurses) and preceptees (interns) reported facing several challenges during their preceptorship programs. These were classified into agential factors, namely, language barriers and lack of readiness to practice, and structural factors, namely, lack of time and lack of direction and role preparation.

#### 3.1.1. Language Barrier

Language barriers hindered the effectiveness of preceptorship programs, as noted by both preceptors and preceptees. They had to communicate in English, which could create a language barrier. Students’ first language was Arabic, and some preceptors found it difficult to speak Arabic despite working in Saudi Arabia for several years. “*We sometimes face difficulties in the language because the Arabic language is our main language.*” (*Intern 1*).

The lack of language skills affected the learning process. The preceptors felt that the preceptees could not understand the in‐depth explanations being provided to them because of their insufficient language skills*:* “*Even if I want to explain more about the procedure or clarify its rationale, it seems that students are not proficient in English.*” (*Preceptor 1*).

#### 3.1.2. Lack of Readiness to Practice

Most interns felt unprepared to practice their clinical skills during their internships and expressed a fear of making mistakes, which underscores a lack of confidence. One preceptee stated “*What if I make a mistake in inserting the cannula and the patient gets hurt; what if I did not do the wound dressing well?*” (*Intern 3*).

Similarly, preceptors pointed out that interns’ lack of knowledge and skills affected their ability to become successful preceptors. Preceptees’ lack of readiness was also echoed by a preceptor: “*I found out during my preceptorship that their* [students’] *practical knowledge or their theoretical knowledge of procedures was insufficient.*” (*Preceptor 1*).

On the other hand, preceptors felt unprepared for their precepting roles and needed professional development to be effective. One preceptor stated “*I should update my knowledge and information related to the specific skills in this field so it will help the project be successful.*”(*Preceptor 3*)*.*

#### 3.1.3. Lack of Time

The shortage of nurses in clinical settings results in a lack of availability in terms of time; this was considered a barrier to interns learning. From the preceptors’ perspective, precepting was time‐consuming, largely because they take on multiple roles as care providers and doing other ward‐related activities. Balancing the supervision of preceptees, clinical teaching, and patient care activities was challenging, especially owing to the preceptor‐to‐preceptee ratio. Nurses stated “*I work as head nurse and also preceptor; this is so difficult*” (*Preceptor 1*) and “*I have difficulty dividing my time equally among the preceptees that I am handling.*” (*Preceptor 2*) Another nurse commented “*I am responsible for many patients simultaneously [the patient-to-nurse ratio is 9:1]*.” (*Preceptor 4*).

From the preceptees’ perspective, the preceptors’ lack of time affected their learning experience. One intern commented “*Actually, I think that our preceptors in hospitals have a lot of work. So, they do not have much time to teach students. I think this will limit the students’ learning experience.*” (*Intern 4).*

#### 3.1.4. Lack of Role Preparation and Direction

The interviews showed that preceptorship programs lacked a clear role and direction regarding the teaching process, expected responsibilities, and evaluation practices. From the preceptors’ perspective, there was no clear pathway for preparing nurses to become preceptors. Some preceptors reported assuming the role without formal orientation or adequate understanding of preceptorship. One preceptor stated “*I became a nurse preceptor without knowing the meaning of preceptorship.*” (*Preceptor 1*). Preceptors also emphasized that training, workshops, and lectures on how to supervise preceptees were inadequate.

From the preceptees’ perspective, the lack of direction was reflected in the absence of guidance and/or supervision during clinical training. Interns reported that they were sometimes given tasks without sufficient explanation or structured supervision. One preceptee stated “*The preceptor gives us the freedom to do a list of tasks rather than go step by step and describe the tasks for us.*” (*intern 3*), while another stated “*Some nurses tell us to give medication to the patient without supervision, which can result in any mistake.*” (*intern* 5).

These findings indicate that a lack of role preparation affected both groups: preceptors lacked formal preparation for their teaching role, while preceptees lacked structured guidance during clinical practice.

### 3.2. Theme 2: Strategies for Improving Preceptorship Programs

Participants from both groups identified several strategies for improving current preceptorship programs. These strategies were grouped into two subthemes: (1) having an organized preceptorship program and (2) having organizational support. Consistent with the interview guide, these strategies reflected participants’ views on how challenges could be addressed and how the current state of preceptorship programs could be improved.

#### 3.2.1. Having an Organized Preceptorship Program

From the preceptors’ perspective, participants emphasized that preceptorship programs should be more structured and clearly organized to support both teaching and learning. Preceptors highlighted the importance of clear guidance, standardization of expectations, and practical resources to support their teaching role. One preceptor suggested the use of a manual to guide preceptors’ responsibilities and evaluation practices: “*If there is a manual, let the preceptor read the manual and follow its steps to enhance the current state of the preceptorship program.*” (*Preceptor 1*).

Another preceptor recommended that preceptees should receive preparatory learning materials before entering the clinical setting: “*Give them like a video before they go to the hospital and assess the feelings they will experience before they practice nursing skills in the real world.*” (*Preceptor 8*)*.* These views indicate that preceptors saw organized preparation, clear direction, and structured educational tools as key elements of a stronger preceptorship program.

From the preceptees’ perspective, interns emphasized the need for a more structured clinical learning experience, particularly one that prioritized supervised hands‐on practice over routine or predominantly task observation. One preceptee stated “*We could only see and not practice or do the procedures. Watching gives some experience, but when we actually do the procedure, it helps the information stay in our minds differently than when we only observe the procedure.*” (*intern 5*)*.*

Preceptees also indicated a need for clearer guidance and guidelines during clinical training, as reflected in their earlier accounts of being left to complete tasks with insufficient explanation. Taken together, these findings suggest that both valued an organized preceptorship approach that clearly outlines expectations, emphasizes practical learning, and provides more direct instructional support during clinical placement.

#### 3.2.2. Having Organizational Support

From the preceptors’ perspective, organizational support was regarded as essential for the success of preceptorship programs. Preceptors emphasized the importance of support from leadership, adequate staffing, and sufficient time allocation for teaching and supervision. One preceptor stated: “*Support from higher authorities is much needed to make this preceptorship program a success.*” (*Preceptor 2*).

Preceptors also emphasized the need for sufficient staffing and a suitable preceptor–preceptee ratio to support more effective supervision and orientation. As one participant explained: “*Having a large enough workforce such as by increasing the number of preceptors in the unit to facilitate the orientation is necessary to establish one-on-one preceptor–preceptee relationships.*” (*Preceptor 2*) Another nurse stated: “*No additional work is added for the preceptor, which means that they are free for training so that they can devote themselves to teaching, evaluating, and follow-up.*” (*Preceptor 5*).

Some preceptors further highlighted the importance of recognition and compensation for the additional workload involved in precepting students. One participant commented: “*Look at the benefits offered to the preceptor, the financial benefits, of course. For example, they should be paid a training allowance.*” (*Preceptor 1*).

Preceptors also highlighted the value of mentorship as part of institutional support, with one participant stating: “*A mentor should be present; they are ‘gold.’ They can advise them [students] on what is proper.*” (*Preceptor 7*)*.* This suggests that participants viewed mentoring support as part of the wider organizational infrastructure needed to strengthen preceptorship.

From the preceptees’ perspective, interns also supported the preceptors’ opinion. They recognized the need for stronger organizational support, particularly in ways that would allow preceptors to spend more time teaching. They linked their learning experience to staffing and workload conditions in the clinical environment. One preceptee commented: “*Actually, I think that our preceptors in hospitals have a lot of work. So, they do not have much time to teach students. I think this will limit the students’ learning experience.*” (*Intern 4*).

Preceptees’ accounts indicate that improving staffing levels, reducing unnecessary workload burdens on preceptors, and creating conditions that allow more consistent supervision would improve their clinical learning experience.

## 4. Discussion

This study sheds light on the challenges of preceptorship programs and potential strategies for their improvement by studying the perspectives of those involved in them. Two main themes emerged from the analysis: one reflecting the challenges related to preceptorship and the other highlighting potential strategies for improving preceptorship.

### 4.1. Challenges of Preceptorship

The challenges faced by preceptors and preceptees are multifaceted and include limited language skills, lack of readiness to practice, lack of time, and lack of direction and role preparation. These findings are consistent with those of previous reports, implying that preceptorship challenges are similar across various settings [20, 21]. The lack of language skills was perceived as a challenge in preceptorship programs. From the preceptors’ viewpoint, even if they wanted to describe clinical concepts in depth, the limited English skills of the preceptees affected their understanding. Language barriers can negatively influence students’ abilities to seek assistance from nursing staff [11]. Although English is integrated into the nursing curriculum in Saudi Arabia, language skills continue to remain a barrier, which may be attributed to the late introduction of English in Saudi schools, thus hindering students’ ability to understand the language well [15, 22].

The lack of readiness to practice their roles was challenging for both preceptors and preceptees. While preceptees lacked confidence in performing specific tasks in a clinical setting, they perceived their preceptors as lacking the necessary knowledge and skills to guide them. A lack of opportunities for practice contributes to low self‐confidence among interns [23]. Nursing interns and newly graduated nurses may experience worry and fear in dealing with real patients; this is attributed to their lack of confidence, as nursing skills are primarily practiced in laboratories rather than in real‐life situations. Therefore, it is crucial for preceptors to possess the required knowledge and skills to help undergraduate students achieve their clinical learning outcomes. Unfortunately, the nurses in our study lacked sufficient knowledge and skills about preceptorship, which affected their readiness to practice preceptorship. Similar findings were reported by a previous study conducted in Saudi Arabia [15]. Professionally, preceptors and preceptees must be prepared for their respective roles [24]; however, none of the nurses in this study had received training. A lack of training may negatively affect not only their role clarity but also their knowledge, skills, and readiness to supervise their preceptees [25].

Imparting knowledge and skills to preceptees requires preceptors to engage with them effectively and deliver constructive feedback [26]. However, the nurses in this study lacked sufficient time to teach and evaluate students’ performance because of the overwhelming patient workload coupled with the shortage of nurses. Previous researchers have also reported that precepting undergraduate students are time‐consuming, specifically because of the multiple roles of nurses and the need to divide time between patient care and supervising students [27]. Preceptors find it challenging to balance the additional task of precepting preceptees with patient care responsibilities, resulting in missed preceptor–preceptee interaction opportunities and, consequently, the underdevelopment of students’ clinical skills. An increased workload makes precepting time‐consuming and cumbersome, affecting nurses’ ability to function effectively [21, 28].

### 4.2. Strategies for Improving Preceptorship

The participants pointed out that imparting training for the role, providing organizational support by decreasing workload, ensuring a larger workforce, providing sufficient time for preceptees, and ensuring recognition through financial rewards are strategies for improving preceptorship. The strategies suggested by the participants have been previously reported by other researchers [20]. Preceptor preparation is crucial to preceptors’ clinical role. Training nurses in the preceptor role emerged as a measure for improving preceptorship programs in our study, which was confirmed by a previous descriptive phenomenological study of nurses and midwives [20].

In addition to preceptor preparation, the findings showed that preceptees also valued a more organized and practical learning experience. Preceptees emphasized the need for clearer instructions, supervised hands‐on practice, and more consistent guidance from preceptors. This indicates that improving preceptorship programs requires attention not only to preceptor training but also to the structure of students’ clinical learning experiences. Organizational support is, therefore, essential for strengthening preceptorship programs. Adequate staffing can reduce the workload placed on preceptors and allow them to spend more time supervising students. Protected teaching time can help preceptors focus on teaching, evaluation, and follow‐up without compromising patient care. Appropriate preceptor‐to‐preceptee ratios can also promote closer supervision and more meaningful clinical learning. In addition, mentorship and recognition of the preceptor role can motivate preceptors and reinforce the value of their contribution to students’ professional development.

Preceptors are traditionally known to function as educators who guide preceptees in developing their clinical knowledge and skills [26]. This is consistent with Bandura’s self‐efficacy theory [29], which posits that students’ knowledge acquisition is aligned with performance achievement. The educational preparation of preceptors has several benefits for students, preceptors, and organizations; previous studies have reported an increased level of confidence in the ability to precept and improved ability to assess the clinical skills of a preceptee [30]. Similarly, improved confidence in precepting nursing students and enhanced preparedness and understanding of the preceptor’s role in assessment were revealed in a recent quasiexperimental study conducted in Saudi Arabia [31]. The nurses in our study also suggested informal training using manuals as a strategy for role preparation. Furthermore, the participants believed that active participation in the learning process would enhance students’ clinical skills, which would require the use of pedagogy for role preparation of both preceptors and preceptees. Therefore, offering nurse preceptor educational programs could positively impact the roles of preceptors [5, 10].

The findings also showed that preceptees valued active participation in clinical learning. Preceptees indicated that they needed more structured opportunities to perform hands‐on tasks under supervision. This finding complements the preceptors’ suggestions for manuals, preparatory materials, and clearer program structure, because both groups emphasized the importance of moving beyond unstructured or mainly theoretical learning experiences.

The nurses in our study fulfilled several roles concurrently, including precepting students, which was perceived as an extra burden. Our findings are supported by the results of a qualitative study using grounded theory among nurses in Australia. The major theme in that study was “the added extra,” indicating the perception of registered nurses that having a preceptee was an additional responsibility in their daily work [32]. To balance the workload appropriately and avoid compromising the additional role of precepting a large number of students, it is important to have an adequate number of preceptors.

Ensuring an adequate preceptor–preceptee ratio to improve preceptorship programs was one of the strategies recommended by the nurses in our study. The high workload in clinical settings, coupled with the shortage of trained preceptors, makes it even more essential for organizations to support preceptors by providing an appropriate preceptor–preceptee ratio. Providing adequate time for preceptors to engage in preceptorship‐related activities was recommended by the nurses in our study as another strategy to improve preceptorship programs. This finding is consistent with that of a previous study reporting precepting undergraduate students as a time‐consuming process [33]. With the immense time preceptors spend on preceptees, they have little time to learn themselves.

The nurses in our study suggested that another strategy that organizations could use to improve preceptorship programs would be recognition through financial rewards for the extra effort and time spent on preceptorship, which is also in line with previous studies [20, 34]. Nevertheless, providing recognition in any form increases nurses’ motivation, interest, and commitment, which can consequently improve their clinical experience [21]. Alternatively, providing free and continuous educational programs on preceptorship could be a motivational factor [20]. Subsequently, nurses could use these hours to renew their Saudi Council licenses.

Overall, the findings suggest that strategies for improving preceptorship programs should address both the structure of the program and the organizational environment in which preceptorship occurs. A more organized preceptorship program can support role clarity, readiness, hands‐on practice, and fair evaluation, which is consistent with previous studies emphasizing the importance of preceptor preparation, educational guidance, communication, and assessment support [26, 30, 31]. Organizational support is also essential, particularly through adequate staffing, protected teaching time, appropriate preceptor–preceptee ratios, mentorship, and recognition of the preceptor role [18, 19, 26, 30, 31]. Integrating these strategies may help address the barriers identified by both preceptors and preceptees and strengthen the overall quality of clinical learning experiences [18, 19, 32].

### 4.3. Strengths and Limitations

The preceptees and preceptors who participated in this study were recruited from different hospitals in Saudi Arabia, thus enhancing the transferability of the findings to similar multicultural contexts. The data generated are essential for nurse educators and decision makers to design suitable and effective preceptorship programs for nursing interns and newly recruited nurses. However, this study faced several limitations.

Although the participants were recruited from different regions of Saudi Arabia, their perceptions toward preceptorship programs may not reflect the actual practice in the selected hospitals. Furthermore, as the interviews were conducted in English, the Arabic‐speaking participants may not have been able to answer the questions perfectly, compared with those whose native language was English. Additionally, there is a dearth of research on preceptors’ and preceptees’ perceptions toward preceptorship in Saudi Arabia, thus limiting us from comparing our findings with other contexts in the country.

### 4.4. Implications and Recommendations

Preceptorship programs are essential for cultivating skilled and confident nurses who can contribute positively to healthcare systems. Implementing effective preceptorship programs can address challenges within the nursing curriculum and profession and enhance overall healthcare outcomes. Thus, based on our findings, educators and policymakers should address the barriers identified by both students and nurses in our study. By doing so, preceptorship can help nursing interns integrate theory into practice, build their confidence and skills, and internalize their profession’s roles and values in a supportive and nurturing environment [35]. Using technology, such as a mobile application preferably augmented by artificial intelligence, can enhance clarity around the roles of both preceptors and preceptees, support the mapping of learning objectives, provide access to supplemental resources, deliver immediate feedback, and facilitate remote follow‐up and collaboration between academic institutions and clinical settings [33]. It can also enhance the achievement of personalized learning pathways and readiness by identifying a preceptee’s specific knowledge gaps and tailor learning materials to their unique needs [34].

The literature does not provide a detailed description of ideal preceptorship programs for interns. Future researchers are recommended to design a standardized preceptorship program for newly recruited nurses and nursing internes. This may include training methods and hours, evaluation methods, number of preceptees per preceptor, mentoring methods and duration, and the matched‐pair design of preceptors and preceptees. It is also recommended that future researchers utilize a longitudinal design to test changes over time and suggest a standardized preceptorship program based on their findings.

## 5. Conclusions

Preceptorship is essential for helping nursing students develop clinical knowledge, confidence, and practical skills as they transition into professional nursing roles. However, this study highlights several barriers faced by both preceptees and preceptors in clinical settings, including language barriers, lack of readiness to practice, lack of time, and unclear direction and role preparation. Improving preceptorship requires both an organized program structure and strong organizational support. Healthcare organizations should support preceptors by ensuring adequate staffing, an adequate number of preceptors for the number of preceptees, and recognition of the preceptor role. Addressing these areas would strengthen the quality of preceptorship programs, support preceptors in their teaching responsibilities, and enhance preceptees’ clinical learning experiences. Future research is warranted to design standardized preceptorship programs that incorporate these strategies and re‐examine their effectiveness in improving clinical education outcomes.

## Author Contributions

All the authors contributed to this study based on the criteria of the International Committee of Medical Journal Editors.

Adnan Innab: conceptualization, methodology, visualization, validation, software, and writing–review and editing.

Juliana Linnette D′Sa: methodology, validation, drafting the work, and writing–review and editing.

Sahar Alshamrani: conceptualization, software, and writing–review and editing.

Salha Alshamari: data curation, investigation, and drafting the work.

Amani Awaji: conceptualization and writing–review and editing,

Nawdhaa Alotaibi: visualization and drafting the work.

Wejdan Shaqiqi: methodology, validation, data curation, and writing–review and editing.

## Funding

This study was funded by the Ongoing Research Funding Program (ORF2026‐837) of King Saud University, Riyadh, Saudi Arabia.

## Disclosure

All authors read and approved the final manuscript.

## Ethics Statement

Institutional Review Board approval was obtained from the Ministry of Health, Saudi Arabia [H‐01‐R‐012], before data collection. This study was conducted according to the principles of the Declaration of Helsinki.

## Conflicts of Interest

The authors declare no conflicts of interest.

## Data Availability

The data that support the findings of this study are available from the corresponding author upon reasonable request.
